# Predictors of non-diagnostic cytology in surgeon-performed ultrasound guided fine needle aspiration of thyroid nodules

**DOI:** 10.1186/s40463-014-0048-0

**Published:** 2014-12-03

**Authors:** Andre Isaac, Caroline C Jeffery, Hadi Seikaly, Hani Al-Marzouki, Jeffrey R Harris, Daniel A O’Connell

**Affiliations:** Division of Otolaryngology-Head and Neck Surgery, University of Alberta, #603-10649 Saskatchewan Drive, Edmonton, AB T6E 6S8 Canada

**Keywords:** Thyroid nodule, FNA, Diagnostic yield, Ultrasound

## Abstract

**Background:**

Fine needle aspiration (FNA) is the standard of care for the diagnostic work-up of thyroid nodules but despite its proven utility, the non-diagnostic rate for thyroid FNA ranges from 6-36%. A non-diagnostic FNA is problematic for the clinician and patient because it can result in repeated procedures, multiple physician visits, and a delay in definitive treatment. Surgeon-performed FNA has been shown to be safe, cost-effective, as accurate as those performed by other clinicians, and has the added benefit of decreasing wait times to surgery. Several studies have examined rates and factors that may be predictive of a non-diagnostic cytology in non-surgeon FNA, but none have evaluated this in surgeon-performed thyroid FNA. If these factors are unique in surgeon-performed vs. non-surgeon performed thyroid FNA, then patients may be more appropriately triaged to FNA by alternate clinicians.

**Objectives:**

The purpose of this study was to determine the rate and factors predictive of a non-diagnostic FNA in surgeon performed ultrasound-guided FNA of thyroid nodules.

**Methods:**

We conducted a retrospective review of all adult patients who underwent thyroid FNA by a staff, fellow, or resident Otolaryngologist at the University of Alberta between January 2011 and June 2013. Factors analyzed included patient factors, thyroid characteristics, nodule characteristics, and surgeon level of training and experience. Univariate and multivariate binary logistic regression analysis were performed.

**Results:**

131 patients (180 nodules) were reviewed. The non-diagnostic rate was 23%. Nodules with predominant cystic component, those less than 1 cm, and resident-performed FNA were associated with non-diagnostic cytology (p = 0.001, p = 0.02, p = 0.04 respectively). A cystic nodule was the only independent predictor of non-diagnostic FNA on multivariate analysis (OR = 4.441, 95% CI [1.785-11.045], p = 0.001).

**Conclusions:**

The rate of non-diagnostic thyroid FNA performed by a surgeon with ultrasound guidance is similar to other clinicians. A cystic nodule is a strong independent predictor of non-diagnostic cytology. Non-cystic nodules may particularly benefit from surgeon-performed thyroid FNA due to the high diagnostic rate and potential for earlier definitive management.

## Introduction

Thyroid nodules are common, with up to 7% of adults demonstrating clinically palpable nodules [1] and up to 70% of adults have evidence of nodules on ultrasound [2]. Fine needle aspiration (FNA) is the standard of care for the diagnostic work-up of thyroid nodules [3] with sensitivity and specificity ranging from 65-98% and 72-100%, respectively [4,5]. Surgeon-performed FNA has been shown to be safe, cost-effective, and shows clinical equipoise compared to those performed by other clinicians. Surgeon performed FNA has the added benefit of potentially decreasing wait times to surgery [6-8]. The decrease in wait times is of particular importance in Canada because a recent survey of Canadian Otolaryngologists revealed a discord between perceived appropriate wait times and actual wait times to thyroid surgery in Canada [9].

Despite its proven utility, the non-diagnostic rate for thyroid FNA ranges from 6-36% [10-16]. A non-diagnostic FNA is problematic for the clinician and patient because it can result in repeated procedures, multiple physician visits, and most importantly a delay in definitive treatment [17]. There is a generalized lack of consensus in Canada on how to manage a non-diagnostic aspirate with reported opinions ranging from repeat FNA to early surgical management [18]. Several studies have examined rates and factors that may be predictive of a non-diagnostic FNA but none have evaluated surgeon-performed thyroid FNA [11,12,19]. If the rate and predictive factors of non-diagnostic cytology in surgeon-performed thyroid FNA are different than that of other clinicians, then certain patients may particularly benefit from early surgeon or non-surgeon referral.

The purpose of this study was to determine the rate and factors that are predictive of a non-diagnostic FNA in surgeon performed ultrasound-guided FNA of thyroid nodules.

## Methods

We conducted a retrospective review of all adult patients who underwent fine needle aspiration biopsy of one or more thyroid nodules at the University of Alberta Hospital Division of Otolaryngology between January 2011 and June 2013. Institutional review board (University of Alberta Health Research Ethics Board Panel B) ethics approval was obtained prior to commencement of the study (HREB: Pro 00041540).

Inclusion criteria were all adults with a diagnostic thyroid ultrasound demonstrating nodules meeting American Thyroid Association (ATA) 2012 guidelines for diagnostic FNA. Patients must have undergone FNA by one of three fellowship trained Head and Neck Oncology and Reconstructive Surgeons, the Head and Neck Oncology fellow or an Otolaryngology-Head and Neck surgery resident. Patients were excluded from the study if their aspirates were lost or improperly processed, or if the pathology or ultrasound reports were unavailable.

FNA biopsies were performed in the clinic under ultrasound guidance, and analyzed by staff cytopathologists at the University of Alberta Hospital according to the Bethesda system for reporting thyroid cytopathology [20]. Patient variables collected included basic demographics such as age, sex, and BMI. Thyroid variables included reported dimensions on ultrasound (a thyroid was considered “enlarged” if one lobe was greater than 6 cm in largest dimension), thyroid function (by most recent TSH at time of biopsy), presence of multinodular goiter, presence of Grave’s disease or thyroiditis, and relevant history of lymphoma or radiation.

Diagnostic thyroid ultrasounds reports were also reviewed to identify nodule variables. All thyroid ultrasounds were interpreted by a staff radiologist at the University of Alberta Hospital. Nodule variables examined included size, number, location (right/left lobe, upper/lower pole, isthmus), vascularity, echogenicity, presence of calcifications, and nodule components (predominantly solid, cystic, or complex). Performer variables included level of training (staff surgeon, fellow, or resident), and the level of experience with FNA. Previous studies have shown that 50 or more FNAs are required to gain proficiency and thus this was used as the cutoff to differentiate between “experienced” and “inexperienced” [21]. Final pathology was classified as diagnostic or non-diagnostic according to the Bethesda system [20].

Descriptive and inferential statistics were performed using SPSS 22. The primary outcome was the rate of non-diagnostic FNA biopsy, and the groups were compared using a two-sample t-test for numerical data, or a chi-squared test for nominal data. Multivariate binary logistic regression was used to determine the relationship between the non-diagnostic rate and each of the patient, thyroid, nodule, and surgeon variables recorded. The significance level was set at p < 0.05.

## Results

152 patients met the inclusion criteria. 21 were excluded due to incomplete data (17 for unavailable ultrasound reports, 2 for unavailable pathology, 1 for improperly reported pathology, and 1 for mislabeling of the aspirate which resulted in it being discarded). 131 patients were included in the data analysis, for a total of 180 nodules aspirated. The overall non-diagnostic rate was 23%. The demographic and patient factors for diagnostic and non-diagnostic FNA are summarized in Table 1. Patient factors were not statistically different between those who had diagnostic and non-diagnostic FNA.

**Table 1:** **Demographics of patients with diagnostic and non-diagnostic thyroid FNA**

		***FNA result***		
***Variable***		***Diagnostic (n = 139)***	***Non-diagnostic (n = 41)***	***p-value***
**Average age**		53.3 (+/-14.3) yrs	57.3 (+/-15.6) yrs	0.20
**Female**		91%	90%	1
**Thyroid function**	Euthyroid	97%	95%	1
	Hyperthyroid	0%	0%	
	Hypothyroid	0%	5%	
**Diagnosis**	Multinodular goiter	35%	42%	0.54
	Grave's disease	0%	0%	
	Hashimoto's	0%	0%	
**Relevant history**	Radiation	2%	4%	1
	Lymphoma	0%	0%	
	Head/neck surgery	0%	0%	

FNA = fine needle aspiration.

Percentages are based on 131 patients, for a total of 180 nodules aspirated (139 diagnostic, 41 non-diagnostic).

No difference was found in mean nodule size, palpability, and location between diagnostic and non-diagnostic aspirates. However, nodule size less than 1 cm and cystic nodules had higher non-diagnostic rates (p = 0.02, and p = 0.001 respectively) (Table 2). Resident-performed FNA was associated with a higher non-diagnostic rate, although only ten FNA were performed by a resident (p = 0.04, Table 3).

**Table 2:** **Thyroid and nodule features in diagnostic and non-diagnostic thyroid FNA**

***Variable***		***Diagnostic (n = 139)***	***Non-diagnostic (n = 41)***	***p-value***
**Thyroid dimensions**	Enlarged	29%	33%	0.66
	Not enlarged	71%	67%	
**Palpable**		77%	64%	0.07
**Nodule size**	Average	23 mm (+/- 13 mm)	19 mm (+/- 15 mm)	0.25
	>4 cm	10%	8%	0.8
	<1 cm	18%	35%	0.02
**Nodule location**	Right	56%	49%	0.38
	Left	44%	51%	
	Upper	24%	18%	0.8
	Lower	55%	50%	0.35
	Isthmus	22%	32%	0.55
**Adverse features**	Calcifications	22%	10%	0.1
	Hypervascularity	49%	35%	0.14
	Hypoechoic	15%	10%	0.59
**Components**	Solid	53%	43%	0.36
	Cystic	11%	30%	0.001
	Complex	37%	28%	0.33

FNA = fine needle aspiration.

“Enlarged” = at least one thyroid lobe greater than 6 cm in greatest dimension on ultrasound.

Percentages are based on 131 patients, for a total of 180 nodules aspirated (139 diagnostic, 41 non-diagnostic).

**Table 3:** **Surgeon and experience factors in diagnostic and non-diagnostic thyroid FNA**

***Variable***		***Number of FNA performed (n = 180)***	***Diagnostic (n = 139)***	***Non-diagnostic (n = 41)***	***p-value***
**Mean number of passes**			2.3	2.1	0.11
**Level of training**	Staff	153	86%	80%	0.44
	Fellow	17	12%	10%	1
	Resident	10	2%	10%	0.04
**Experience**	>50		14%	20%	0.44

FNA = fine needle aspiration.

Percentages are based on 131 patients, for a total of 180 nodules aspirated (139 diagnostic, 41 non-diagnostic).

Multivariate analysis with binary logistic regression showed that a cystic nodule was the only predictor of a non-diagnostic aspirate, with an odds ratio of 4.441 (95% CI [1.785-11.045], p = 0.001, see Table 4).

**Table 4:** **Multivariate analysis by binary logistic regression of predictors of non-diagnostic cytology in surgeon-performed thyroid FNA**

***Variable***	***Odds ratio***	***95% CI***	***Sig.***
Palpability	0.743	0.264-2.096	0.575
Size <1 cm	1.900	0.641-5.637	0.247
Cystic	4.441	1.785-11.045	0.001
Resident-performed	4.497	0.849-30.228	0.074

Based on 131 patients, for a total of 180 nodules aspirated (139 diagnostic, 41 non-diagnostic).

## Discussion

Ultrasound guided FNA has become the standard of care for the diagnostic workup of thyroid nodules over the past fifteen years, with several studies demonstrating increased diagnostic yield, safety, and cost-effectiveness over traditional FNA [22,23]. Ultrasound guided FNA performed by a surgeon has gained particular popularity in the recent literature, as a method to further decrease surgical wait-time as well as increase patient convenience and satisfaction due to a decreased number of specialist referrals [6-8,24,25]. However, a non-diagnostic aspirate can necessitate the involvement of other clinicians including radiologists, cytopathologists and endocrinologists. This can result in a delay in diagnosis and definitive management, which has prognostic significance, since patients with a non-diagnostic FNA have a significantly higher rate of all types of thyroid cancer [10]. The overall risk of malignancy in non-diagnostic thyroid FNA is also high, ranging from 2-14% [26-28]. Undetected carcinoma can delay treatment by up to 28.2 months, resulting in higher rates of vascular and capsular invasion, and a higher likelihood of persistent disease [17]. Current ATA guidelines recommend that two non-diagnostic thyroid FNAs in the presence of clinical suspicion of malignancy warrants diagnostic hemi-thyroidectomy [29]; however, some have argued that this approach could result in increased rates of exposure to surgical risk, unnecessary complications, and results in permanent hypothyroidism in up to 47% of patients [30]. Thus, ideally, thyroid nodules that are more likely to yield non-diagnostic cytology by a surgeon should be referred to alternate clinicians early in their diagnostic work up, and vice versa.

The population studied here was representative of a typical patient population seen by most thyroid surgeons functioning at a tertiary referral center, with a strong predominance of females and a wide range of ages. This is strengthened by the fact that we reviewed consecutive patients referred to the clinic from any source, without exclusion of any particular patient subpopulations. Thus, we believe our results to be widely applicable to the majority of surgeons performing thyroid surgery in North America.

Our results demonstrated an association between nodule size <1 cm and non-diagnostic cytology in univariate analysis, although this was not found in multivariable analysis. Choi et al. and Moon et al. found similar results with respect to nodule size, with nodule less than 5 mm being significantly associated with a non-diagnostic FNA in radiologist-performed aspirates [19,31]. Nodules less than 5 mm in size were not studied here due to the small number of nodules that met this criterion.

According to our results, the only reliable independent predictor of a non-diagnostic aspirate in surgeon-performed FNA is a predominantly cystic nodule. This is in keeping with findings by Alexander et al. and Grani et al. who both found that a cystic nodule was strongly associated with a higher likelihood of non-diagnostic cytology in endocrinologist-performed thyroid FNA [11,12]. Similarly, studies based on radiologist-performed thyroid FNA have found that nodules with more than 50% cystic portion increased the likelihood of a non-diagnostic result [19,31].

Although resident-performed FNA had a higher non-diagnostic rate in our study, no firm associations can be made due to the relatively small number of resident-performed aspirates included in this study. In addition, this may simply reflect the fact that residents perform fewer numbers of aspirates throughout their training. Indeed, Houlton et al. has argued that clinician volume is a strong predictor of non-diagnostic FNA [21]. Our study failed to demonstrate a similar finding, although likely due to the relatively small sample size.

When we compare our non-diagnostic predictors with those of other clinicians in the literature, endocrinologist and radiologist-performed aspirates are also more likely to be non-diagnostic in cystic nodules and small nodules. However, studies in other clinicians have also found that hypoechogenicity [11,19,31], and female sex [32] were associated with a higher non-diagnostic rate, which we did not find in surgeon-performed thyroid FNA. Patients with these features may particularly benefit from early surgeon referral.

Notably, we did not study the utility of on-site cytopathologist evaluation of aspirates, as this is not routinely performed at our institution. Our group is examining the utility of on-site evaluation, with data collection for that study currently under way.

In the future, we plan to design a similar study with prospectively collected data, and a standardized radiological protocol for reporting features on thyroid ultrasound that can further elucidate more subtle predictors of non-diagnostic cytology and help to improve the diagnostic rate at our and other centers. This may also be used to design a randomized controlled trial to directly compare surgeon-performed FNA with that of other clinicians, which has not been examined previously in a prospective manner. This may help to further inform patient triage decisions.

## Conclusions

The rate of non-diagnostic thyroid FNA performed by a surgeon with ultrasound guidance is similar to that of other clinicians. A predominantly cystic nodule is a strong independent predictor of non-diagnostic cytology in surgeon-performed thyroid FNA. Patients with solid or non-cystic thyroid nodules may particularly benefit from surgeon-performed thyroid FNA due to the high diagnostic rate and potential for earlier definitive management.

## References

[1] Hambleton C, Kandil E (2013). Appropriate and accurate diagnosis of thyroid nodules: a review of thyroid fine-needle aspiration. Int J Clin Exp Med.

[2] Goldfarb M, Gondek S, Solorzano C, Lew JI (2011). Surgeon-performed ultrasound can predict benignity in thyroid nodules. Surgery.

[3] Hegedüs L, Clinical practice (2004). The thyroid nodule. N Engl J Med.

[4] Porterfield JR, Grant CS, Dean DS, Thompson GB, Farley DR, Richards ML, Reading CC, Charboneau JW, Vollrath BK, Sebo TJ (2008). Reliability of benign fine needle aspiration cytology of large thyroid nodules. Surgery.

[5] Ogilvie JB, Piatigorsky EJ, Clark OH (2006). Current status of fine needle aspiration for thyroid nodules. Adv Surg.

[6] Bohacek L, Milas M, Mitchell J, Siperstein A, Berber E (2012). Diagnostic accuracy of surgeon-performed ultrasound-guided fine-needle aspiration of thyroid nodules. Ann Surg Oncol.

[7] Al-azawi D, Mann GB, Judson RT, Miller JA (2012). Endocrine surgeon-performed US guided thyroid FNAC is accurate and efficient. World J Surg.

[8] Bhatki AM, Brewer B, Robinson-Smith T, Nikiforov Y, Steward DL (2008). Adequacy of surgeon-performed ultrasound-guided thyroid fine-needle aspiration biopsy. Otolaryngol Head Neck Surg.

[9] Brake M, Moore P, Taylor SM, Trites J, Murray S, Hart R (2013). Expectantly waiting: a survey of thyroid surgery wait times among Canadian otolaryngologists. J Otolaryngol Head Neck Surg.

[10] Coorough N, Hudak K, Jaume JC, Buehler D, Selvaggi S, Rivas J, Sippel R, Chen H (2013). Nondiagnostic fine-needle aspirations of the thyroid: is the risk of malignancy higher?. J Surg Res.

[11] Grani G, Calvanese A, Carbotta G, D'Alessandri M, Nesca A, Bianchini M, Del Sordo M, Fumarola A (2013). Intrinsic factors affecting adequacy of thyroid nodule fine-needle aspiration cytology. Clin Endocrinol (Oxf).

[12] Alexander EK, Heering JP, Benson CB, Frates MC, Doubilet PM, Cibas ES, Marqusee E (2002). Assessment of nondiagnostic ultrasound-guided fine needle aspirations of thyroid nodules. J Clin Endocrinol Metab.

[13] Baier ND, Hahn PF, Gervais DA, Samir A, Halpern EF, Mueller PR, Harisinghani MG (2009). Fine-needle aspiration biopsy of thyroid nodules: Experience in a cohort of 944 patients. AJR Am J Roentgenol.

[14] Degirmenci B, Haktanir A, Albayrak R, Acar M, Sahin DA, Sahin O, Yucel A, Caliskan G (2007). Sonographically guided fine-needle biopsy of thyroid nodules: The effects of nodule characteristics, sampling technique, and needle size on the adequacy of cytological material. Clin Radiol.

[15] Kim DW, Lee EJ, Kim SH, Kim TH, Lee SH, Kim DH, Rho MH (2009). Ultrasound-guided fine-needle aspiration biopsy of thyroid nodules: Comparison in efficacy according to nodule size. Thyroid.

[16] Marqusee E, Benson CB, Frates MC, Doubilet PM, Larsen PR, Cibas ES, Mandel SJ (2000). Usefulness of ultrasonography in the management of nodular thyroid disease. Ann Intern Med.

[17] Yeh MW, Demircan O, Ituarte P, Clark OH (2004). False-negative fine-needle aspiration cytology results delay treatment and adversely affect outcome in patients with thyroid carcinoma. Thyroid.

[18] Parks NE, Taylor SM, Trites JR, Hart RD (2012). Management of goitre and small nodule disease by Canadian otolaryngologists. J Otolaryngol Head Neck Surg.

[19] Moon HJ, Kwak JY, Kim EK, Kim MJ (2011). Ultrasonographic characteristics predictive of nondiagnostic results for fine-needle aspiration biopsies of thyroid nodules. Ultrasound Med Biol.

[20] Cibas ES, Ali SZ (2009). The Bethesda system for reporting thyroid cytopathology. Am J Clin Pathol.

[21] Houlton JJ, Sun GH, Fernandez N, Zhai QJ, Lucas F, Steward DL (2011). Thyroid fine-needle aspiration: does case volume affect diagnostic yield and interpretation?. Arch Otolaryngol Head Neck Surg.

[22] Danese D, Sciacchitano S, Farsetti A, Andreoli M, Pontecorvi A (1998). Diagnostic accuracy of conventional versus sonography-guided fine-needle aspiration biopsy of thyroid nodules. Thyroid.

[23] Carmeci C, Jeffrey RB, McDougall IR, Nowels KW, Weigel RJ (1998). Ultrasound-guided fine-needle aspiration biopsy of thyroid masses. Thyroid.

[24] Robitschek J, Straub M, Wirtz E, Klem C, Sniezek J (2010). Diagnostic efficacy of surgeon-performed ultrasound-guided fine needle aspiration: a randomized controlled trial. Otolaryngol Head Neck Surg.

[25] Karadeniz Cakmak G, Emre AU, Tascilar O, Gultekin FA, Ozdamar SO, Comert M (2013). Diagnostic adequacy of surgeon-performed ultrasound-guided fine needle aspiration biopsy of thyroid nodules. J Surg Oncol.

[26] Hryhorczuk AL, Stephens T, Bude RO, Rubin JM, Bailey JE, Higgins EJ, Fox GA, Klein KA (2012). Prevalence of malignancy in thyroid nodules with an initial nondiagnostic result after ultrasound guided fine needle aspiration. Ultrasound Med Biol.

[27] Jo VY, Vanderlaan PA, Marqusee E, Krane JF (2011). Repeatedly nondiagnostic thyroid fine-needle aspirations do not modify malignancy risk. Acta Cytol.

[28] Richards ML, Bohnenblust E, Sirinek K, Bingener J (2008). Nondiagnostic thyroid fine-needle aspiration biopsies are no longer a dilemma. Am J Surg.

[29] Cooper DS, Doherty GM, Haugen BR, Kloos RT, Lee SL, Mandel SJ, Mazzaferri EL, McIver B, Pacini F, Schlumberger M, Sherman SI, Steward DL, Tuttle RM, American Thyroid Association (ATA) Guidelines Taskforce on Thyroid Nodules and Differentiated Thyroid Cancer (2009). Revised American Thyroid Association management guidelines for patients with thyroid nodules and differentiated thyroid cancer. Thyroid.

[30] Balentine CJ, Domingo RP, Patel R, Laucirica R, Suliburk JW (2013). Thyroid lobectomy for indeterminate FNA: not without consequences. J Surg Res.

[31] Choi YS, Hong SW, Kwak JY, Moon HJ, Kim EK (2012). Clinical and ultrasonographic findings affecting nondiagnostic results upon the second fine needle aspiration for thyroid nodules. Ann Surg Oncol.

[32] Parikh PP, Allan BJ, Lew JI (2013). Sex variability of fine-needle aspiration reliability in the diagnosis of malignancy in thyroid nodules ≥4 cm. Am J Surg.

